# Integrated multi-omics analysis identifies *ENY2* as a predictor of recurrence and a regulator of telomere maintenance in hepatocellular carcinoma

**DOI:** 10.3389/fonc.2022.939948

**Published:** 2022-08-04

**Authors:** Jian-Hua Li, Yi-Feng Tao, Cong-Huan Shen, Rui-Dong Li, Zheng Wang, Hao Xing, En-Si Ma, Hong-Yuan Xue, Quan-Bao Zhang, Zhen-Yu Ma, Zheng-Xin Wang

**Affiliations:** ^1^ Department of General Surgery, Huashan Hospital, Fudan University, Shanghai, China; ^2^ Department of Critical Care Medicine, Huashan Hospital, Fudan University, Shanghai, China; ^3^ Department of General Surgery, Comprehensive Breast Health Center, Ruijin Hospital, Shanghai Jiao Tong University School of Medicine, Shanghai, China

**Keywords:** hepatocellular carcinoma, recurrence risk, prognostic factor, *ENY2*, telomere maintenance

## Abstract

Hepatocellular carcinoma (HCC) is the most common type of primary liver cancer and has a high recurrence rate. Accurate prediction of recurrence risk is urgently required for tailoring personalized treatment programs for individual HCC patients in advance. In this study, we analyzed a gene expression dataset from an HCC cohort with 247 samples and identified five genes including *ENY2*, *GPAA1*, *NDUFA4L2*, *NEDD9*, and *NRP1* as the variables for the prediction of HCC recurrence, especially the early recurrence. The Cox model and risks score were validated in two public HCC cohorts (GSE76427 and The Cancer Genome Atlas (TCGA)) and one cohort from Huashan Hospital, which included a total of 641 samples. Moreover, the multivariate Cox regression analysis revealed that the risk score could serve as an independent prognostic factor in the prediction of HCC recurrence. In addition, we found that *ENY2*, *GPAA1*, and *NDUFA4L2* were significantly upregulated in HCC of the two validation cohorts, and *ENY2* had significantly higher expression levels than another four genes in malignant cells, suggesting that *ENY2* might play key roles in malignant cells. The cell line analysis revealed that ENY2 could promote cell cycle progression, cell proliferation, migration, and invasion. The functional analysis of the genes correlated with *ENY2* revealed that ENY2 might be involved in telomere maintenance, one of the fundamental hallmarks of cancer. In conclusion, our data indicate that ENY2 may regulate the malignant phenotypes of HCC *via* activating telomere maintenance.

## Introduction

Liver cancer is a malignancy contributing to approximately 8.3% of total cancer-related deaths worldwide, according to the Global Cancer Observatory (GLOBCAN) 2020 estimates (1). Hepatocellular carcinoma (HCC), the most common form of primary liver cancer, is highly heterogeneous, and the exploration of molecular indicators of HCC prognosis has developed around altered gene expressions and compositions of intratumor immune cells (2–4). However, due to the heterogeneous nature of HCC, there is a lack of markers to predict recurrence in every HCC patient. Still, we seek to understand HCC pathogenesis and metastasis from different perspectives.

Tumor recurrence after hepatic resection is regarded as an important indicator of long-term survival in HCC. Clinically, spleen stiffness measurement (SSM) is an independent non-invasive marker of portal hypertension (PH), and according to a recent study, SSM is the sole marker significantly associated with late HCC recurrence (5). Meanwhile, indicators of HCC recurrence on a genetic level have been explored. The differentially expressed genes (DEGs) between tumor and non-cancerous samples were often investigated, and researchers have identified several DEGs such as BUB1, CDC20, KIF20A, RACGAP1, and CEP55, which could be involved in the recurrence of HCC (5). Altered expression levels of two DNA methylation-driven genes, namely, SPP1 and LCAT, were reported to be associated with early recurrence and unfavorable outcomes, and their expressions in serum samples might serve as potential non-invasive markers for recurrence surveillance (6). Also, upregulated expression of exosomal microRNA miR-638 could be related to HCC recurrence, as it played a tumor-suppressive role in HCC *via* affecting the cell cycle and cell migration (7). Still, markers to efficiently monitor and predict HCC recurrence are currently lacking.

The Spt-Ada-GCN5-acetyltransferase (SAGA) complex shares a close relationship with multiple posttranslational histone modifications, and *ENY2*, a protein-coding gene, is a critical component of the SAGA complex (8, 9). Two distinct enzymatic modules within the SAGA complex, namely, histone acetylation (HAT) and deubiquitination (DUB) modules, are associated with transcription activation and mRNA export, thereby regulating specific gene expressions (10, 11). It is worth noticing that increased ENY2 expression was observed in patients with multiple cancers, as compared to healthy controls (12). ENY2 participates in the regulation of histone H2B monoubiquitination (H2Bub1) levels and is essential for maintaining the H2B/HB2ub1 balance (13). When such a balance is broken, DNA repair machinery would be disturbed, which may further lead to tumor development and progression (14). In the present study, we aimed to conduct bioinformatics analyses to identify the recurrence-related genes, derive a risk score to evaluate the recurrence risk in HCC, and explore the functionalities of those genes to interpret their associations with HCC recurrence.

## Materials and methods

### Genome-wide gene expression data acquisition

We obtained the gene expression datasets of GSE14520 (15) and GSE76427 (16) from the Gene Expression Omnibus (GEO, https://www.ncbi.nlm.nih.gov/geo/), and the HCC gene expression data of The Cancer Genome Atlas (TCGA) (17) from University of California, Santa Cruz (UCSC) Xena (https://xena.ucsc.edu/). The RNA sequencing (RNA-seq) and microarray data were normalized to fragment per kilo million (FPKM) and Robust Multichip Average (RMA)-based log2 intensity, respectively. The summarized clinical characteristics of HCC patients from the three cohorts could be seen in **Table 1**.

**Table 1:** The summary of clinical characteristics for the four HCC cohorts.

Factors	GSE14520	GSE76427	TCGA	Huashan
TNM stage (%)				
I	38.87%	47.83%	46.09%	16.67%
II	31.58%	30.43%	23.18%	52.56%
III	27.43%	18.26%	22.91%	30.77%
IV	0%	2.61%	1.35%	0.00%
NA	2.02%	0.87%	6.47%	0.00%
BCLC stage (%)				
0	8.10%	3.48%	/	9.62%
A	61.54%	64.35%	/	40.38%
B	9.72%	24.35%	/	28.85%
C	11.74%	7.82%	/	21.15%
NA	8.90%	0%	/	0%
Gender (male)	85.43%	80.87%	67.39%	89.61%
Age (median/SD)	50/11	64/13	61/14	52/10
Recurrence (%)	55.06%	41.74%	45.11%	44.87%
Early recurrence (%)	69.85%	81.25%	83.92%	77.14%
Tumor size (%)				
>5 cm	35.63%	/	/	37.18%
<5 cm	61.94%	/	/	62.17%
NA	3.53%	/	/	0.75%
Multinodular (%)	21.05%	/	/	53.20%
Differentiation (%)				
Well	/	/	/	42.31%
Poor	/	/	/	55.13%
NA	/	/	/	2.56%

SD, standard deviation; NA, not available; HCC, hepatocellular carcinoma; TCGA, The Cancer Genome Atlas; BCLC, Barcelona Clinic Liver Cancer.

### Differential gene expression analysis

The R limma package was employed to identify the differentially expressed genes. To reduce the false-positive rates, the *p*-values were adjusted by the Bonferroni method. The adjusted *p*-value< 0.05 and log2 fold change >1 were considered statistically significant.

### Single-cell RNA sequencing data

The single-cell RNA-seq data were downloaded from GEO with accession GSE125449 using the cell annotation by a previous study (18). The nine HCC samples were retained for our analysis. The count matrix was preprocessed and normalized. The principal component analysis was used to reduce the high dimensions. Graph-based clustering was used to cluster the cell types. All these procedures were conducted in the R Seurat package (19).

### Identification of recurrence-related genes

The genes associated with HCC recurrence-free survival (RFS) were considered the recurrence-related genes. Specifically, the association between RFS and gene expression was tested by the log-rank test. Notably, the expression for each gene was discretized by using the median as a threshold. The multivariate model was built based on the selected gene signatures. The Cox model was built by the R survival package (20).

### The gene set enrichment analysis

To interpret the functionality of a given gene set, we conducted a gene set overrepresentation enrichment analysis, which employed the hypergeometric test to calculate the statistical significance, to identify the biological pathways (BP) (21). The ORA was implemented in the R clusterProfiler package (22).

### Cell culture and transfection

The Roswell Park Memorial Institute (RPMI)-1640 medium supplemented with 10% fetal bovine serum (Gibco) and 1% penicillin–streptomycin was used to culture the HCC cell lines, including MHCC-97H, SK-Hep1, and Hep3B. The cells were incubated at 37°C with 5% CO_2_. For cell transfection, the shRNA especially binding ENY2 RNA (shRNA 1-3, ENY2-shRNA, **Table S1**), the negative control (Ctrl), Ctrl-shRNA, empty vector (Ctrl-OE), and vector-ENY2 (ENY2-OE) were obtained from GenePharma Co., Ltd (Shanghai, China). The cells in the logarithmic phase were transfected by Lipofectamine 2000 (Thermo Fisher Scientific). The shRNA sequences and the plasmid for ENY2 overexpression are listed in **Tables S1**, **S2**, respectively.

### Quantitative real-time PCR and Western blotting

Total RNA was prepared using TRIzol reagent (Invitrogen) following the guidance. The cDNA was synthesized by 5xTransScript^®^ All-in-One SuperMix and used for qPCR analysis following the manufacturer’s instructions. The cDNA samples were diluted to a 10% concentration and used as templates for qPCR analysis (ABI StepOnePlus). The primers are listed in **Table S2**. The protein expression was quantified by Western blotting. Briefly, the proteins were firstly isolated from the cells using radioimmunoprecipitation assay (RIPA) lysis buffer. Subsequently, the isolated proteins were quantified by the bicinchoninic acid (BCA) assay kit (Beyotime Biotechnology, China). Every 40 μg of total protein was electrophoresed on 10% sodium dodecyl sulfate (SDS)–polyacrylamide gel and then transferred to polyvinylidene fluoride (PVDF) membranes, which were incubated with primary antibodies against ENY2. A secondary antibody was added and incubated for 2 h at 37°. GAPDH gene products were used as the internal controls for both RNA and protein quantification.

### Cell proliferation assay

We performed Cell Counting Kit 8 (CCK8) assays to detect cell proliferation in 96-well plates with 3–5 × 10^3^ cells/well. Following the manual, we detected the absorbance at 450 nm using a microplate reader (Biotek, EPOCH2).

### Cell cycle analysis

HCC cell lines were collected, washed after 72 h, and added with 75% ethanol for fixation. The resuspended cells were then incubated with 2.5 μl of 10 mg/ml RNase at 37°C for 10 min. Subsequently, 5 μl of 10 mg/ml propidium iodide (PI) was used to treat the DNA for 30 min at 4°C without light. The flow cytometer (FC500, Beckman Coulter) was used to monitor the DNA content, and data were collected by ModFit LT 3.3 software.

### Transwell assay

The cell migration and invasion assays were conducted using transwell chambers (8-μm pore size; Millipore), and chambers were coated with Matrigel. The upper chamber was planted with the cells (5 × 10^5^ cells) with 48 h of transfection. The lower chamber was filled with 400 μl of medium containing 20% fetal bovine serum (FBS). The migrated/invaded cells were fixed with 4% paraformaldehyde for 30 min. Subsequently, 0.1% crystal violet was used to stain the fixed cells for another 30 min. Cells were observed randomly in three fields of view under a 100× microscope.

### Participants

From February 2018 to October 2020, a total of 156 patients who were diagnosed with HCC without extrahepatic metastasis and underwent radical resection were recruited from the Huashan Hospital affiliated to Fudan University in Shanghai, China. All the patients were followed up until October 2021 or recurrence. Participants who were diagnosed with HCC combined with other malignant tumors, had a history of malignant tumors, died within 1 month after surgery, or had incomplete follow-up information were excluded from this study. Chest computed tomography (CT) and liver enhancement magnetic resonance imaging (MRI) were performed every 3 months within 2 years after surgery. Confirmation of suspicious liver lesions was achieved by MRI or enhancement liver CT combined with alpha-fetoprotein (AFP) level. Systemic positron emission tomography CT was used to detect and assess systemic metastasis, if necessary. Recurrence was defined as the emergence of clinical, radiological, and/or pathologic diagnosis of a tumor from a previous origin locally or distantly. The clinical characteristics are summarized in **Table 1**. Written informed consent for publication was obtained from all participants. The current study was approved by the Huashan Hospital affiliated to the Fudan University Ethics Committee (Ethical number: KY2021-856).

### Tissue microarray and immunohistochemistry

Tissue samples were prepared, preserved through paraffin embedding (PE), and mounted onto 3-aminopropyltriethoxysilane-coated slides. The hydrogen peroxide/methanol solution was used to dewax and block the samples. The formalin-fixed paraffin-embedded (FFPE) specimens were used to obtain the representative tumor areas. The samples were pressure-cooked in 0.08% citrate buffer for 20 min for antigen retrieval. The dilution that rendered optimal sensitivity and specificity was determined by titering the antibodies against the normal tissues before it was used on the tissue section. Sequential incubations were used to visualize the staining results. The same way was used to treat the negative controls without adding the primary antibody. Two pathologists assessed the immunohistochemistry (IHC) staining independently without prior knowledge of the patient characteristics. Image analysis was performed using Image Pro-Plus software. The same claybank color was selected as the unified criterion for judging the positivity of all immunohistochemistry pictures by using Image-Pro Plus 6.0. Each picture was analyzed to obtain the cumulative integrated optical density (IOD) of positive signals.

### Statistical methods

The comparisons were conducted by analysis of variance (ANOVA), Student’s *t*-test, or Wilcoxon rank-sum test in R programming software. All experiments were replicated three times, and the data are visualized as the mean value ± standard deviation. *p*< 0.05 was considered statistically significant.

## Results

### Construction of recurrence-based Cox model and derivation of risk score in hepatocellular carcinoma

**Figure 1:**
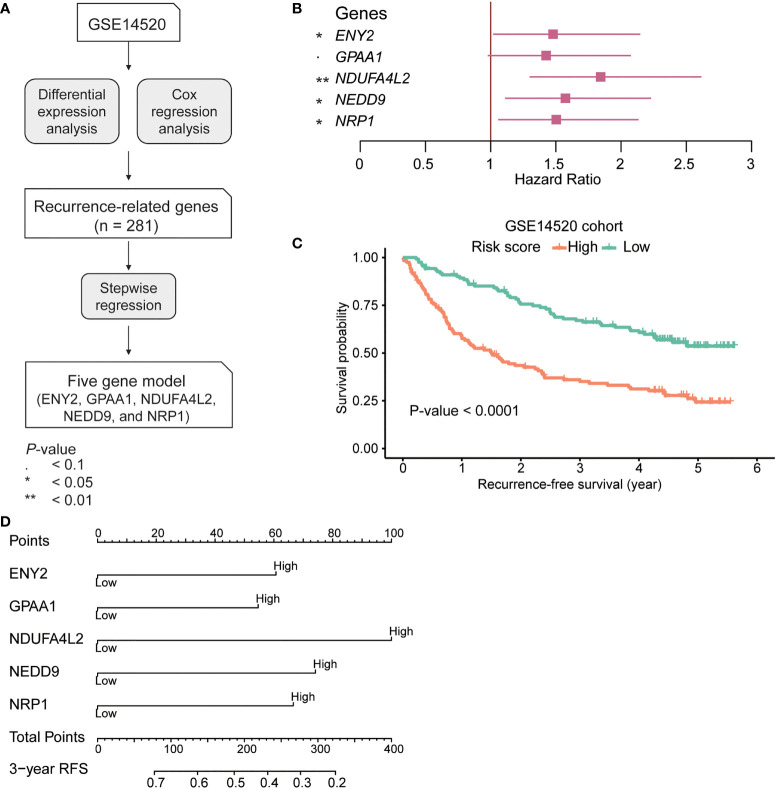
The development and construction of Cox proportional hazard regression model for hepatocellular carcinoma (HCC) recurrence prediction. **(A)** The workflow for the identification of the recurrence-related gene signatures. **(B)** The hazard ratio and 95% confidence interval of the five gene signatures in the recurrence-related Cox model. **(C)** The Kaplan–Meier curves for recurrence-free survival (RFS) of the two risk groups in the GSE14520 cohort (training). The orange and green curves represent the high-risk and low-risk groups, respectively. The 3-year RFS-based nomogram for the five signature genes. **(D)** The signature gene-based nomogram for HCC recurrence prediction. The symbol * and ** represent P-value < 0.05 and 0.01, respectively.

To identify the recurrence-related genes in HCC, we analyzed a gene expression dataset from GEO with accession GSE14520 (n = 247). Specifically, we identified 281 recurrence-related genes by differential gene expression analysis and Cox regression analysis (**Figure 1A**, Wilcoxon rank-sum test, adjusted *p*-value< 0.001; log-rank test, *p*-value< 0.05). Moreover, to narrow down the gene list, we built a multivariate Cox model based on those genes and conducted the stepwise regression analysis on this model. Ultimately, we identified five genes, including *ENY2*, *GPAA1*, *NDUFA4L2*, *NEDD9*, and *NRP1*, which are anti-correlated with RFS (**Figure 1B**). To quantitatively evaluate the risk of recurrence in HCC, we estimated the coefficients of the five genes in the multivariate Cox model, and we derived the risk score using the following equation:


Risk score = −1.0840 + 0.3711 * ENY2 + 0.3342 * GPAA1 + 0.6116 * NDUFA4L2 + 0.4534 * NEDD9 + 0.4073 * NRP1

Furthermore, we divided the samples in the GSE14520 cohort into high-risk and low-risk groups using the median risk score as the threshold. Shorter RFS and higher recurrence rate were observed in the high-risk group as compared with the low-risk group (**Figure 1C**; RFS, 16.4 vs 51.5 months; recurrence rate, 41.42% vs 25.07%, *p*-value< 0.0001). Furthermore, the 3-year RFS-based nomogram also illustrated the relative contribution of the five signature genes to RFS prediction in HCC (**Figure 1D**). In summary, the risk score was highly correlated with HCC recurrence.

### Independent validation of the recurrence-based Cox model and risk score

**Figure 2:**
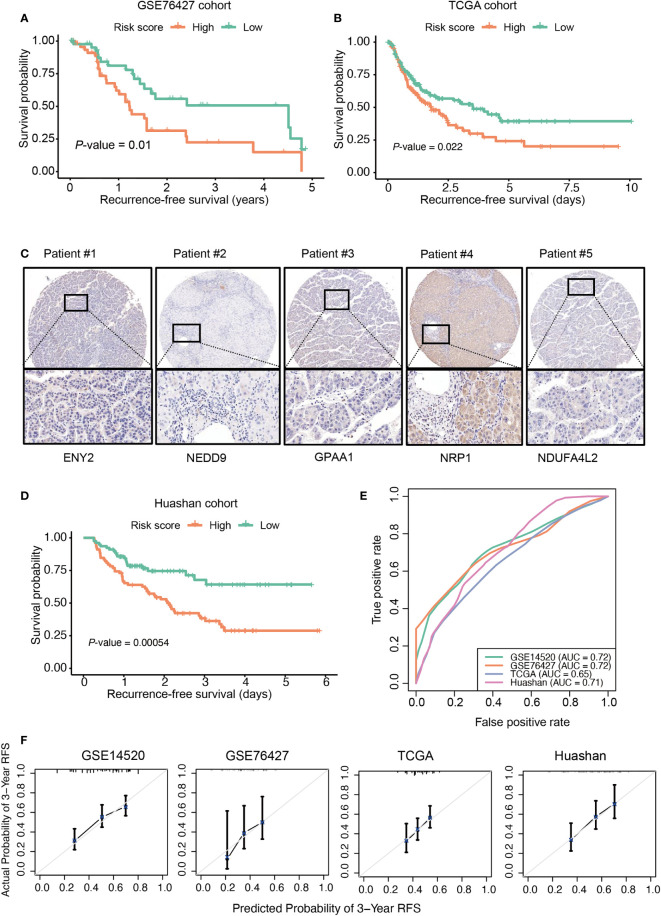
Validation of the recurrence risk score in three independent cohorts. The Kaplan–Meier curves in the three validation cohorts, including GSE76427 **(A)**, The Cancer Genome Atlas (TCGA) **(B)**, The orange and green curves represent the high-risk and low-risk groups, respectively. **(C)** The representative cases with positive immunohistochemical staining of ENY2, NEDD9, GPAA1, NRP1, and NDUFA4L2 in the Huashan cohort (magnification ×200). **(D)** The Kaplan–Meier curves in Huashan cohort. **(E)** The receiver operating characteristic (ROC) curves for the prediction of recurrence by the risk score at 3 years in the hepatocellular carcinoma (HCC) cohorts. **(F)** The calibration curves show the consistency between the predicted probability of 3-year recurrence-free survival (RFS) and the actual probability of 3-year RFS.

To validate the performance of the recurrence-based Cox model and risk score in HCC recurrence prediction, we collected two HCC cohorts of public gene expression datasets from Gene Expression Omnibus (accession: GSE76427, n = 115) and TCGA (n = 371). As with the samples in the training cohort (GSE14520), the samples in the two validation cohorts were also divided into high-risk and low-risk groups. There was a significant difference of RFS between the high-risk and low-risk groups in both of the validation cohorts (**Figures 2A, B**; GSE76427 (RFS, 0.655 vs 0.785 years; recurrence rate, 53.70% vs 35.18%) and TCGA (RFS, 1.44 vs 1.85 years; recurrence rate, 49.68% vs 40.63%), log-rank test: *p*-value = 0.01 and 0.022). In addition, we also collected 156 HCC samples from our institute (Huashan cohort) and conducted an IHC analysis on those samples (**Figure 2C**). Likewise, we also predicted the risk scores for those samples and divided them into high-risk and low-risk groups. Consistently, longer RFS and low recurrence rate were observed in the low-risk group as compared with the high-risk group (**Figure 2D**; RFS, 1.96 vs 2.05 years; recurrence rate, 62.82% vs 26.92%, log-rank test, *p*-value = 0.00054). Furthermore, we also evaluated the model performance by receiver operating characteristic (ROC) and calibration curves at 3-year RFS. Consistently, the AUC value of our model reached 0.7 in both the training and validation cohorts (except the TCGA cohort) (**Figure 2E**). The predicted 3-year RFS showed high consistency with the actual 3-year RFS (**Figure 2F**). These results indicated that the recurrence-based Cox model was robust, and the risk score could efficiently predict HCC recurrence.

### Independence of the risk score in the prediction of hepatocellular carcinoma recurrence

**Figure 3:**
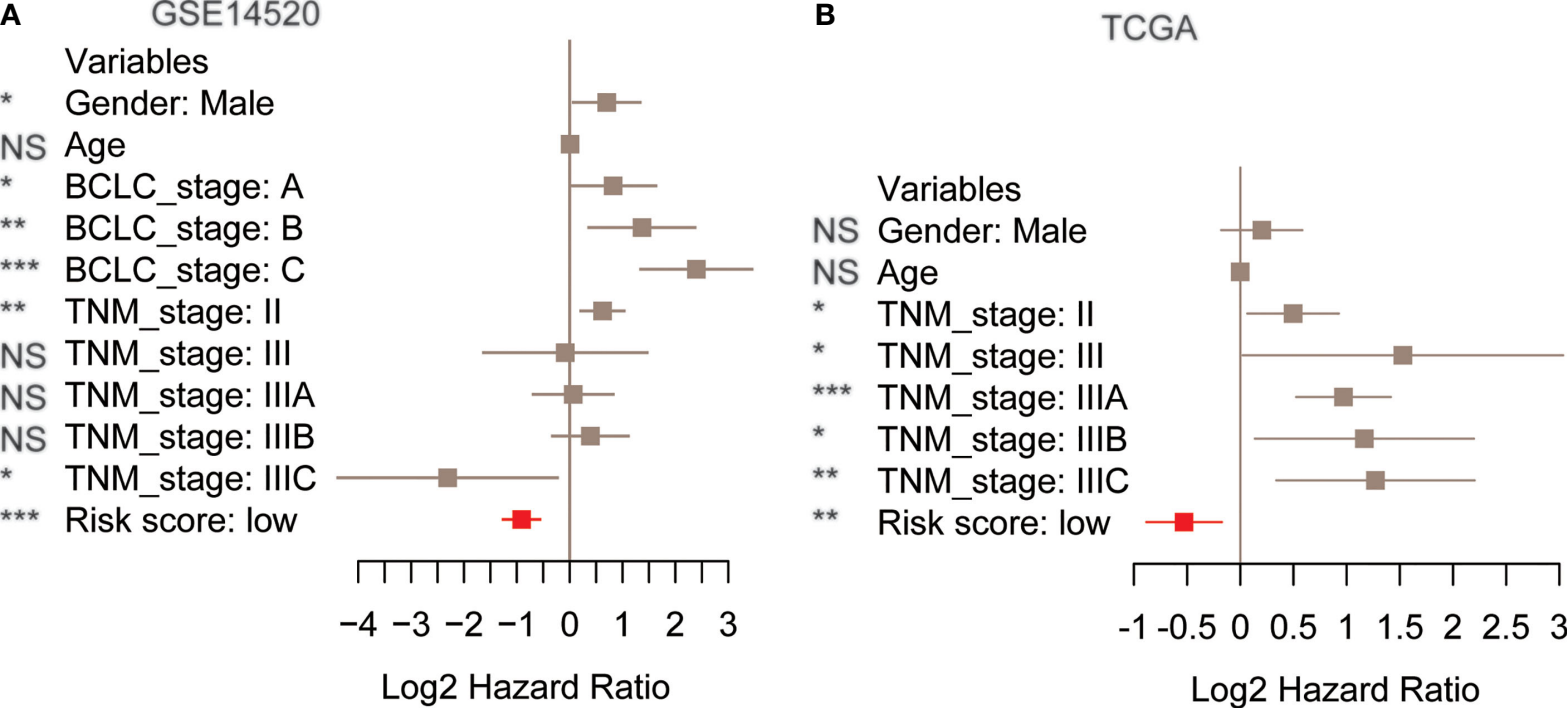
The performance of risk score in the multivariate Cox model using clinical factors as variables. The hazard ratio and 95% confidence interval of the risk score and clinical factors in the multivariate Cox models. The training **(A)** and The Cancer Genome Atlas (TCGA) cohorts **(B)**. The red box represents the risk score. *p*-value< 0.1, *p-value< 0.05, **p-value< 0.01, ***p-value< 0.001. NS, not significant.

To demonstrate the risk score as an independent prognostic factor in the prediction of HCC recurrence, we built a multivariate Cox model based on the risk score/stratification and some prognostically relevant clinical factors like age, gender, TNM stage, and Barcelona Clinic Liver Cancer (BCLC) stage for each cohort. The multivariate Cox regression analysis revealed that the risk score was still statistically significant in the Cox models of the training, TCGA, and GSE76427 cohorts (**Figures 3A, B** and **Table S3**), indicating that the risk score might serve as an independent factor in the prediction of HCC recurrence.

### ENY2 is highly expressed in hepatocellular carcinoma tissues and malignant cells of tumor microenvironment

**Figure 4:**
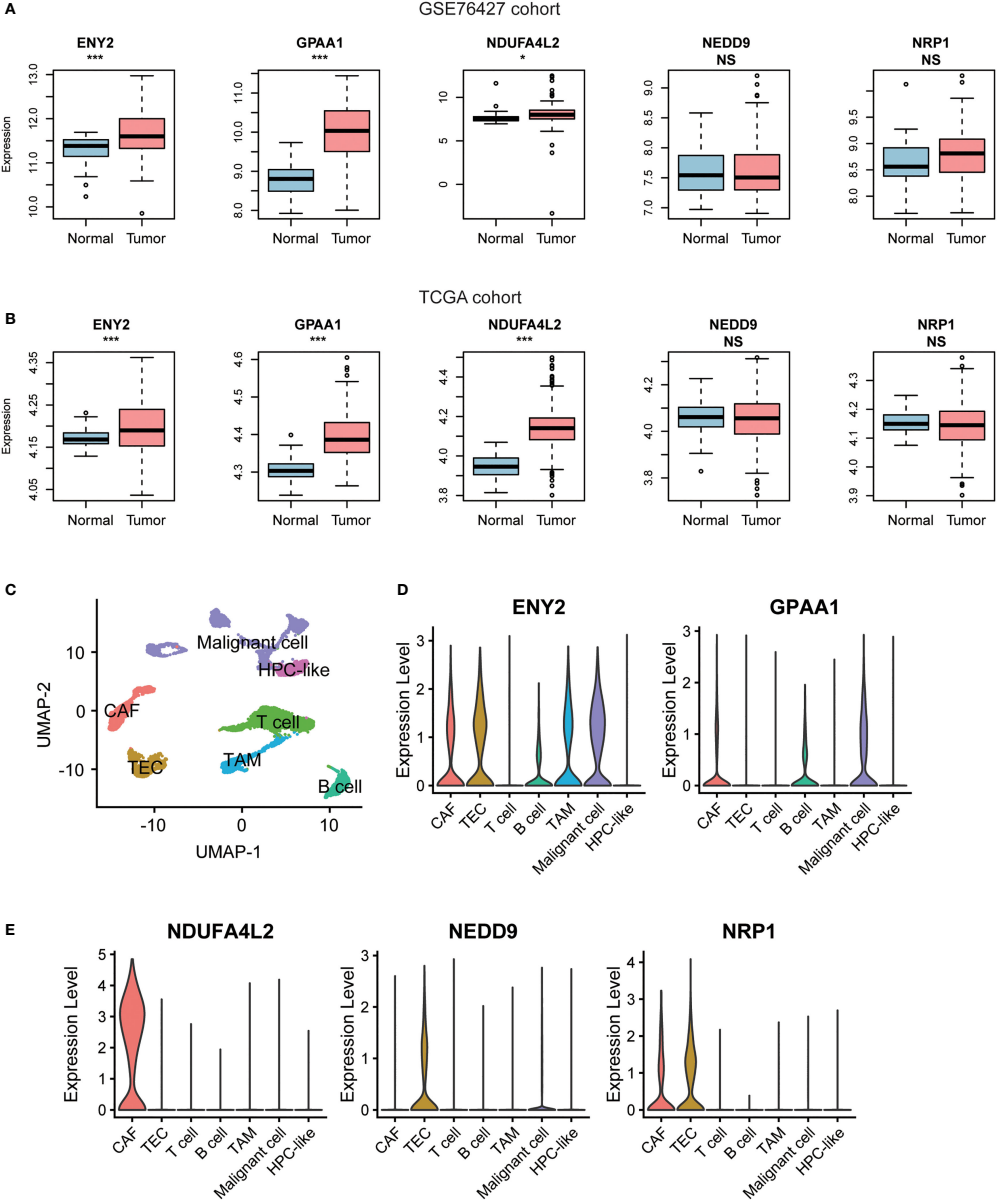
The gene expression patterns of the five recurrence-related genes in hepatocellular carcinoma (HCC) tissues and adjacent normal tissues. The differential gene expression levels of the five recurrence-related genes between HCC and adjacent normal tissues from GSE76427 **(A)** and The Cancer Genome Atlas (TCGA) cohorts **(B)**. The red and blue boxes represent the HCC and adjacent normal tissues, respectively. **(C)** The Uniform Manifold Approximation and Projection (UMAP) for dimension reduction for the cells from 19 liver cancers of GSE125449 cohort. The two genes (*ENY2* and *GPAA1*) highly expressed in malignant cells **(D)** and three genes (*NDUFA4L2*, *NEDD9*, and *NRP1*) highly expressed in non-malignant cells **(E)**. *p-value< 0.05, **p-value< 0.01, ***p-value< 0.001. NS, not significant.

To further investigate the expression of the five recurrence-related genes in HCC tissues and tumor microenvironment (TME), we tested whether those genes were upregulated in the HCC tissues of the two validation cohorts. Consistently, we found that ENY2, GPAA1, and NDUFA4L2 were significantly upregulated in HCC of the two validation cohorts (**Figures 4A, B**). Moreover, we also collected single-cell RNA-seq data of nine HCC samples from GEO (accession: GSE125449) and identified seven major cell types including malignant cell, hepatic progenitor cell (HPC-like), T cell, B cell, cancer-associated fibroblast (CAF), tumor-associated macrophage (TAM), and tumor-associated endothelial cell (TEC) within the TME (**Figure 4C**). Moreover, ENY2 and GPAA1 were expressed in multiple cell types including CAF, TEC, TAM, and/or tumor cells (**Figure 4D**). In addition, NDUFA4L2, NEDD9, and NRP1 were specifically expressed in CAF and/or TEC (**Figure 4E**). Notably, ENY2 had significantly higher expression levels than another four genes in malignant cells (**Table S4**, Wilcoxon rank-sum test, *p*-value< 0.05), suggesting that ENY2 might play key roles in malignant cells. As 77.58% (308/397) of recurrent tumors occurred within 24 months after surgery (**Table 1**, GSE14520 (the training cohort), 69.85%; TCGA, 83.92%; GSE76427, 81.25%; and Huashan, 77.14%), we then investigated the ability of ENY2 to predict the early and late recurrences of HCC. Particularly, ENY2 was closely associated with early recurrence but was weakly correlated with late recurrence (**Figure S1**), suggesting that ENY2 might predict the early recurrence of HCC better than late recurrence.

### ENY2 promotes cell proliferation, migration, and invasion

**Figure 5:**
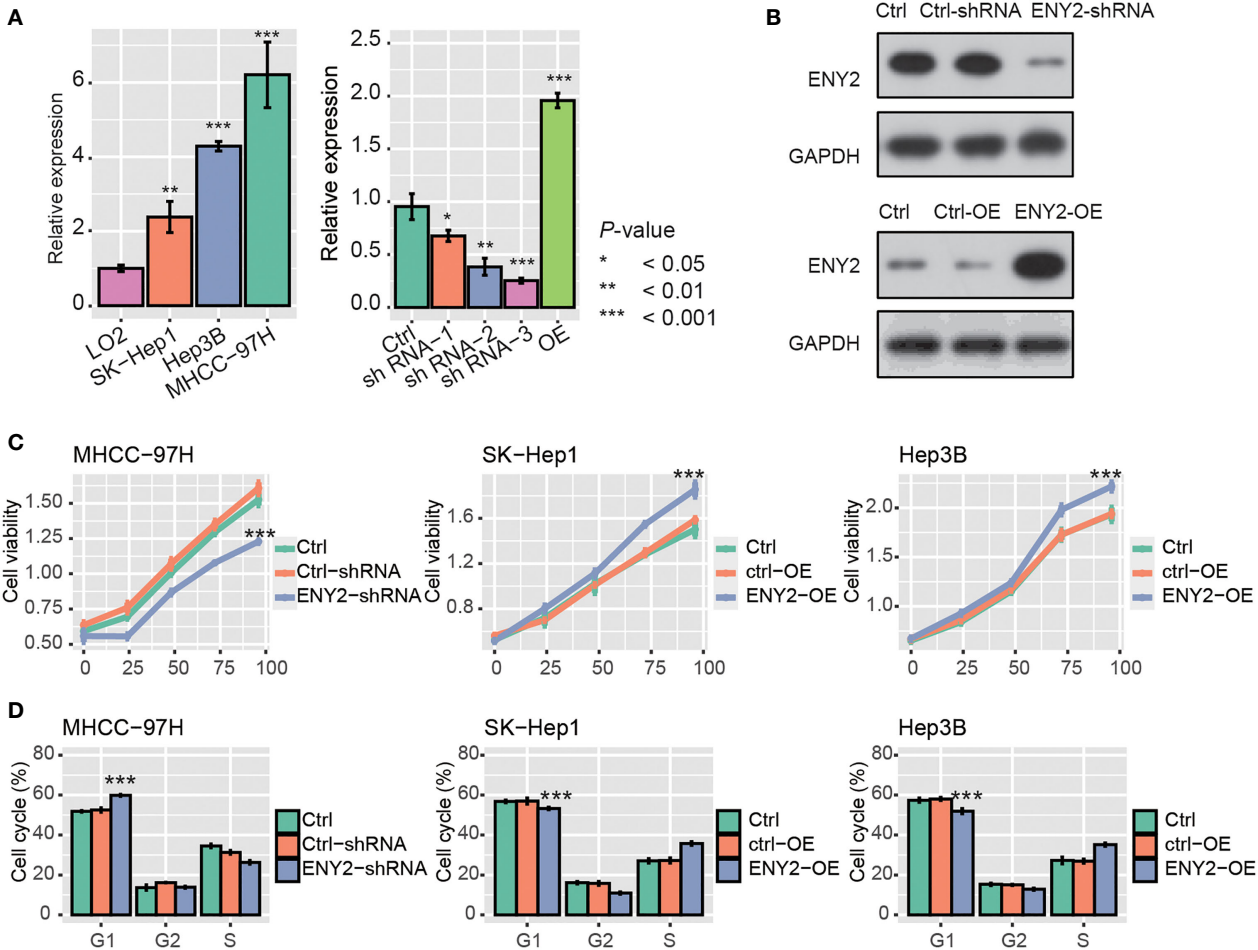
ENY2 silence or overexpression blocked or enhanced cell proliferation and cell cycle progression. **(A)** The RNA expression level of ENY2 in three hepatocellular carcinoma (HCC) cell lines (MHCC-97H, SK-Hep1, and Hep3B) and the efficacy of shRNAs and overexpression vector in 293T cells. **(B)** The efficacy of shRNAs and overexpression vector in 293T cells at protein level by Western blotting assay. **(C)** Cell Counting Kit 8 (CCK8) assay was conducted to determine the cell proliferation ability in HCC cell lines transfected with shRNAs, Ctrl, Ctrl-shRNA, ENY2-overexpression (OE), and Ctrl-OE. **(D)** The proportion of cells transfected with shRNAs, Ctrl, Ctrl-shRNA, ENY2-overexpression (OE), and Ctrl-OE at cell cycle phases G1, G2, and S. *p-value< 0.05, **p-value< 0.01, ***s*p*-value< 0.001.

As ENY2 was highly expressed in malignant cells of HCC, we then investigated whether ENY2 affected the malignant phenotypes of HCC. Firstly, the qPCR analysis revealed that ENY2 RNA expression was higher in HCC cell lines, as compared with the normal hepatocyte cell line, LO2 (**Figure 5A**, *p*-value< 0.05). Moreover, MHCC-97H had a higher expression of ENY2 than SK-Hep1, and the shRNA-3 (ENY2-shRNA) and ENY2-vector (ENY2-OE) could efficiently inhibit and promote the RNA expression levels of ENY2 and were used for ENY2 knockdown or overexpression (**Figure 5A**, *p*-value< 0.05). Consistently, the ENY2 protein expression was also significantly inhibited and enhanced by the knockdown and overexpression (**Figure 5B**). Subsequently, we knocked down or overexpressed ENY2, and we observed the cell proliferation levels in the HCC cell lines. The cell proliferation was significantly decreased by the shRNA treatment in MHCC-97H after 24 h, as compared with Ctrl and Ctrl-shRNA (**Figure 5C**), and SK-Hep1 and Hep3B with ENY2 overexpression (ENY2-OE) exhibited enhanced cell proliferation as compared with controls (Ctrl) (**Figure 5C**). The cell cycle assay showed that knockdown of ENY2 contributed to the increase of cells in the G1 phase and decrease of the cells in the S phase in MHCC-97H cells, while overexpression of ENY2 resulted in the decrease of cells in the G1 phase and increase of the cells in the S phase in SK-Hep1 and Hep3B cells (**Figures 5D**). These results demonstrated that ENY2 could regulate HCC cell proliferation.

**Figure 6:**
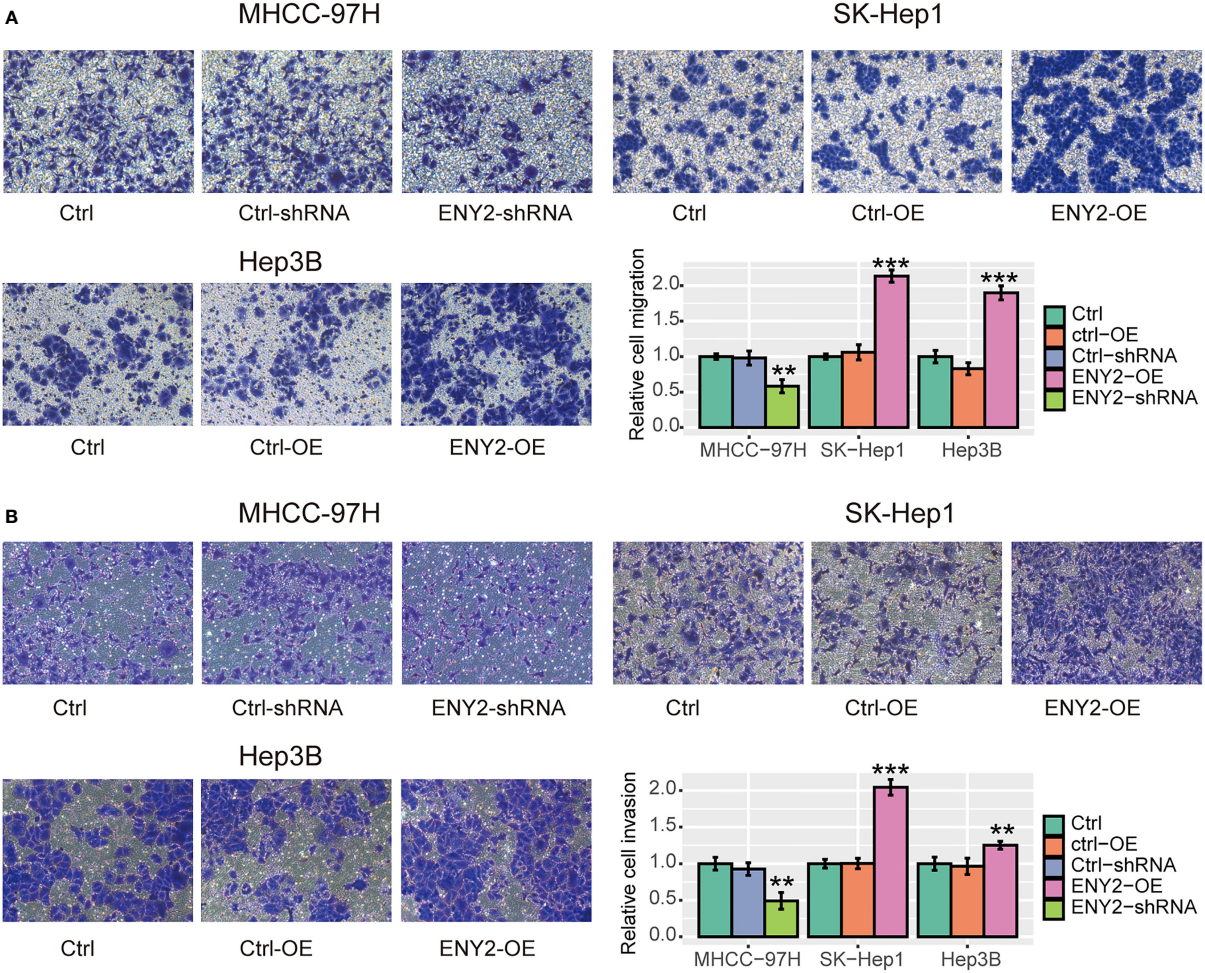
The impact of ENY2 expression on migratory and invasive abilities of hepatocellular carcinoma (HCC) cell lines. Transwell assays were used to measure the migration **(A)** and invasion **(B)** ability of each group. The histogram shows the quantified data (bottom right). *p-value< 0.05, **p-value< 0.01, ***p-value< 0.001.

Furthermore, we performed a transwell assay to assess the impact of ENY2 on HCC metastasis *in vitro*. Specifically, we found that knockdown of ENY2 could significantly suppress the migratory ability and invasiveness of MHCC-97H cells, while overexpression of ENY2 obviously promoted the migratory ability and invasiveness of SK-Hep1 and Hep3B cells (**Figures 6A, B**, *p*-value< 0.05). These results disclosed that ENY2 could significantly affect HCC cell migration and invasion, thereby potentially promoting HCC metastasis.

### ENY2 is associated with telomere maintenance in hepatocellular carcinoma

**Figure 7:**
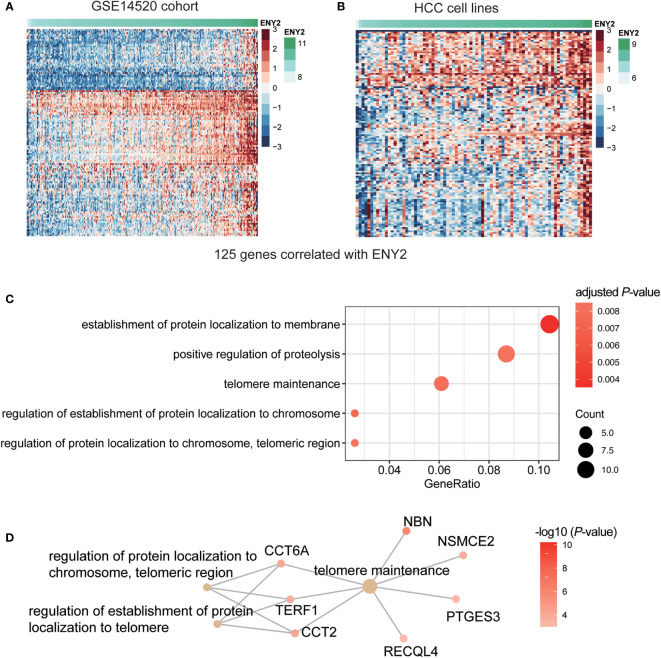
The dysregulated genes and pathways in hepatocellular carcinoma (HCC) with high expression of ENY2. The gene expression patterns of 125 genes highly correlated with ENY2 in training cohort **(A)** and HCC cell lines **(B)**. **(C)** The biological pathways are enriched by the ENY2-related genes. **(D)** The key components involved in telomere maintenance.

To further unveil the molecular mechanism of ENY2 in the regulation of malignant phenotype in HCC, we predicted the biological pathways that ENY2 might be involved. Specifically, we divided the HCC samples from the three HCC cohorts into high (ENY2-hi) and low (ENY2-lo) ENY2 expression groups using the median of ENY2 as the threshold. We identified a total of 125 genes jointly upregulated in the ENY2-hi group of the three cohorts by differential gene expression analysis (**Figure 7A** and **Figure S2**). To further verify the co-expression of ENY2 with the 125 genes, this analysis was also conducted in 79 HCC cell lines, and upregulation of those genes was also observed in the cell lines with high ENY2 expression (**Figure 7B**). The gene set enrichment analysis (GSEA) revealed that the upregulated genes were significantly enriched in the establishment of protein localization to membrane, positive regulation of proteolysis, and telomere maintenance (**Figure 7C**, hypergeometric test, adjusted *p*-value< 0.05). In accordance with this finding, ENY2 has been reported to regulate telomere maintenance (23). Remarkably, CCT6A, NBN, TERF1, NSMCE2, PTGES3, and RECQL4 were identified as the key components of telomere maintenance (**Figure 7D**), suggesting that ENY2 might regulate telomere maintenance through these proteins.

## Discussion

HCC is the most common type of primary liver cancer and has a high recurrence rate. Accurate prediction of recurrence risk is of importance for tailoring personalized treatment programs for individual HCC patients in advance. In this study, we analyzed a gene expression dataset from an HCC cohort with 247 samples and identified a gene list that differentially expressed between HCC and adjacent normal tissues and associated with HCC recurrence. Ultimately, we identified five genes including *ENY2*, *GPAA1*, *NDUFA4L2*, *NEDD9*, and *NRP1* as the variables in the multivariate Cox model. Notably, ENY2 was identified as a prognostic signature for lung metastasis in triple-negative breast cancer (24). Moreover, ENY2, one of the mRNA transcription and export complex subunits, was also observed to have a higher frequency of copy number alterations (CNAs) and a higher mRNA expression level in ovarian cancer (25), indicating that ENY2 might act as a cancer driver gene. GPAA1, a posttranslational glycosylphosphatidylinositol (GPI) anchor attachment, has been reported to regulate some well-recognized cancer driver genes such as c-Myc (26) and EGFR/ERBB2 (27). In addition, *NDUFA4L2*, *NEDD9*, and *NRP1* were identified as prognostic or diagnostic biomarkers in HCC (28–30). To validate the performance of the recurrence-based Cox model and risk score in HCC recurrence prediction, we tested the performance in three datasets including two public datasets (GSE76427 and TCGA cohorts) and one IHC dataset (Huashan cohort). Longer RFS and lower recurrence rates were observed in the low-risk group as compared with the high-risk group (**Figure 2C**, log-rank test, *p*-value = 0.00054), indicating that the recurrence-based Cox model was robust and the risk score could efficiently predict HCC recurrence. Moreover, the multivariate Cox regression analysis revealed that the risk score could serve as an independent prognostic factor in the prediction of HCC recurrence.

In addition, we found that ENY2, GPAA1, and NDUFA4L2 were significantly upregulated in HCC of the two validation cohorts, and ENY2 had significantly higher expression levels than another four genes in malignant cells, suggesting that ENY2 might play key roles in malignant cells. The cell line analysis revealed that ENY2 could promote cell cycle progression, cell proliferation, migration, and invasion. In accordance with this finding, ENY2 was important for cellular proliferation and tumor growth (12). The functional analysis of the genes correlated with ENY2 revealed that ENY2 might be involved in telomere maintenance, one of the fundamental hallmarks of cancer (31). These results suggested that ENY2 might regulate the malignant phenotypes of HCC *via* activating telomere maintenance.

However, the present study still had some limitations. For example, the single-cell RNA-seq might be unable to detect the genes of hepatocyte malignant cells or quantify them with low abundance. The disparity of NRP1 and NDUFA4L2 expression between single-cell RNA-seq data and immunohistochemical staining might be caused by the technical defects in scRNA-seq. Secondly, the five signature genes need to be validated in a larger cohort. Thirdly, the detailed molecular mechanism of ENY2 in the regulation of telomere maintenance will be explored in our further study, which will greatly improve our understanding of how ENY2 affects HCC recurrence.

## Data availability statement

The datasets presented in this study can be found in online repositories. The names of the repository/repositories and accession number(s) can be found in the article/**Supplementary Material**.

## Ethics statement

The studies involving human participants were reviewed and approved by Huashan Hospital Affiliated to the Fudan University Ethics Committee. The patients/participants provided their written informed consent to participate in this study.

## Author contributions

Z-XW conceived and designed the experiments. J-HL, Y-FT, C-HS, and R-DL acquired the data, related materials, and analysis tools. J-HL conducted the experiments and analyzed the data. J-HL, Y-FT, and C-HS wrote the paper. ZW, Z-XW, HX, E-SM, H-YX, Q-BZ, and Z-YM revised the manuscript. All authors read and approved the final manuscript.

## Funding

This study was supported by grants from the National Natural Science Foundation of China (81873874 and 82071797), Clinical Research Plan of SHDC (No. SHDC2020CR2021B and No. SHDC2020CR5012), Foundation of Science and Technology Commission of Shanghai (No. 20Y11908500), Foundation of Shanghai Health Commission (No. 201940032), and Academician Shusen Lanjuan Talent Fundation.

## Conflict of interest

The authors declare that the research was conducted in the absence of any commercial or financial relationships that could be construed as a potential conflict of interest.

## Publisher’s note

All claims expressed in this article are solely those of the authors and do not necessarily represent those of their affiliated organizations, or those of the publisher, the editors and the reviewers. Any product that may be evaluated in this article, or claim that may be made by its manufacturer, is not guaranteed or endorsed by the publisher.

## Supplementary material

Supplementary Figure 1 The association of ENY2 with early and late recurrence of HCC.

Supplementary Figure 2 The gene expression patterns of 125 genes highly correlated with ENY2 in GSE76427 and TCGA cohorts.
