# Supplementary material for: Integrated multi-omics analysis identifies ENY2 as a predictor of recurrence and a regulator of telomere maintenance in hepatocellular carcinoma
Source: Front Oncol. 2022 Aug 4;12:939948. doi: 10.3389/fonc.2022.939948 (PMC9386066; doi:10.3389/fonc.2022.939948)
Supplement: Supplementary file 1 [file Table_1.docx]

**Table S1. Oligomeric single-stranded DNA sequence of Human ENY2 (NM_020189) Easy shRNA**

| Oligomeric single-stranded DNA | sequence 5 ′ to 3 ′ |
| --- | --- |
| ENY2-shRNA-1-1 | 5'-GATCCGCCAGCCTTTAAGATTGAATTATTCAAGAGATAATTCAATCTTAAAGGCTGGTTTTTTG-3' |
| ENY2-shRNA-1-2 | 5'-AATTCAAAAAACCAGCCTTTAAGATTGAATTATCTCTTGAATAATTCAATCTTAAAGGCTGGCG-3' |
| ENY2-shRNA-2-1 | 5'-GATCCGCACACTGTAAAGAGGTAATTTTCAAGAGAAATTACCTCTTTACAGTGTGCTTTTTTG-3' |
| ENY2-shRNA-2-2 | 5'-AATTCAAAAAAGCACACTGTAAAGAGGTAATTTCTCTTGAAAATTACCTCTTTACAGTGTGCG-3' |
| ENY2-shRNA-3-1 | 5'-GATCCGCAGATGAGAGCAGCGATTAATTCAAGAGATTAATCGCTGCTCTCATCTGCTTTTTTG-3' |
| ENY2-shRNA-3-2 | 5'-AATTCAAAAAAGCAGATGAGAGCAGCGATTAATCTCTTGAATTAATCGCTGCTCTCATCTGCG-3' |
