# Supplementary material for: Integrated multi-omics analysis identifies ENY2 as a predictor of recurrence and a regulator of telomere maintenance in hepatocellular carcinoma
Source: Front Oncol. 2022 Aug 4;12:939948. doi: 10.3389/fonc.2022.939948 (PMC9386066; doi:10.3389/fonc.2022.939948)
Supplement: Supplementary file 2 [file Table_2.docx]

**Table S2. Human ENY2 (NM_020189) gene lentiviral expression plasmid**

| NAME | pCDH-CMV-hENY2(NM_020189)-EF1A-GFP-T2A-Puro |
| --- | --- |
| Vector | pCDH-CMV-MCS-EF1A-GFP-T2A-Puro |
| Primer-F | 5’-attcgaatttaaatcggatccGCCACCATGGTGGTTAGCAAGATGAACAAA-3’ |
| Primer-R | 5’ gatcgcagatccttcgcggccgcTTAAAGGCTGGCATGCTGAGC-3’ |
