# Supplementary figures and images for: Integrated multi-omics analysis identifies ENY2 as a predictor of recurrence and a regulator of telomere maintenance in hepatocellular carcinoma
Source: Front Oncol. 2022 Aug 4;12:939948. doi: 10.3389/fonc.2022.939948 (PMC9386066; doi:10.3389/fonc.2022.939948)

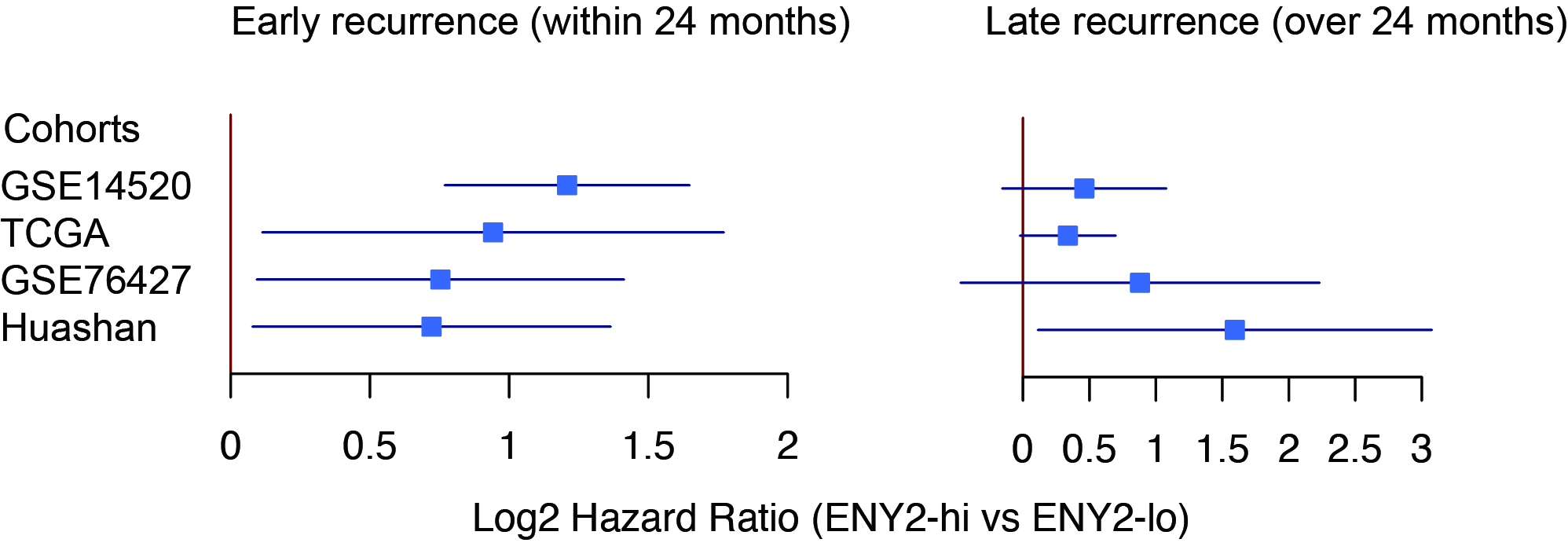

Supplement: Supplementary Figure 1 — The association of ENY2 with early and late recurrence of HCC. [file Image_1.tif]

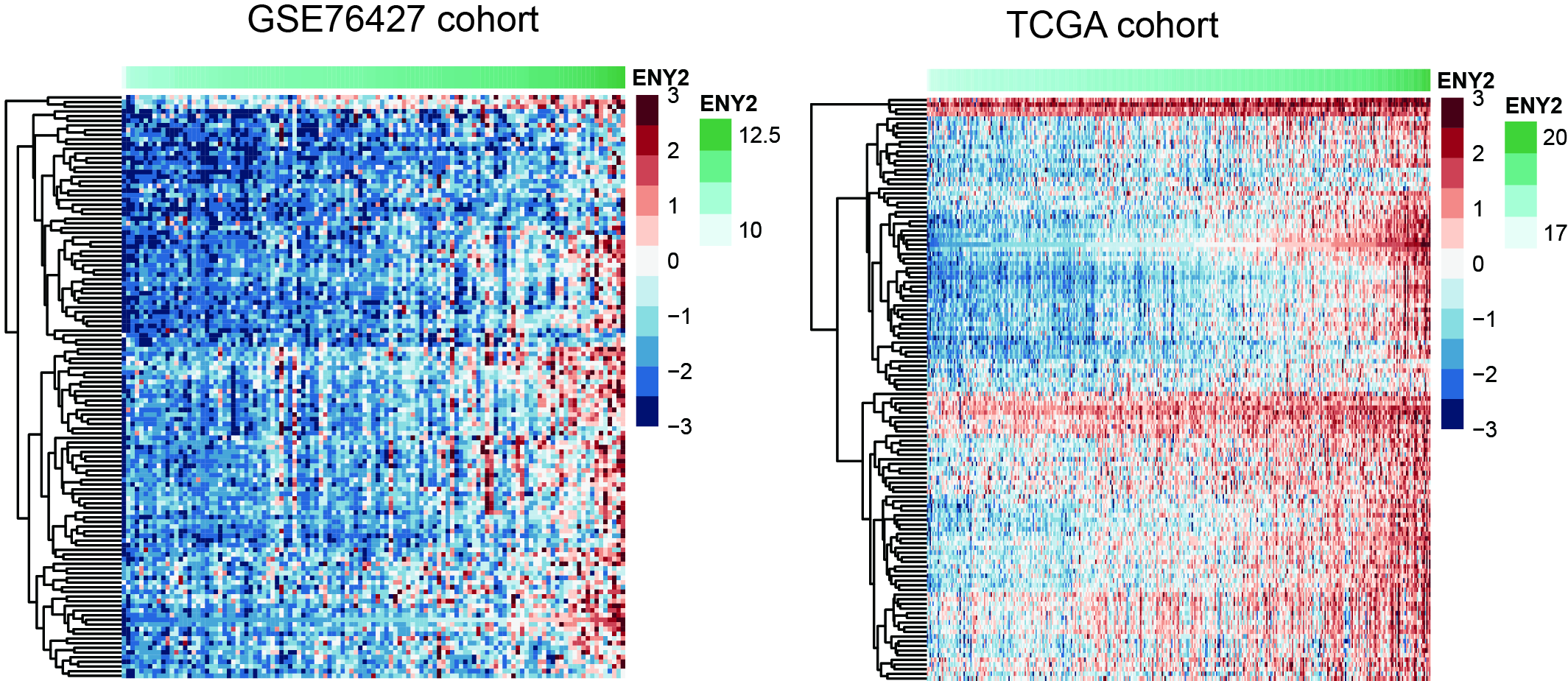

Supplement: Supplementary Figure 2 — The gene expression patterns of 125 genes highly correlated with ENY2 in GSE76427 and TCGA cohorts. [file Image_2.tif]
